# Smoking particles enhance endothelin A and endothelin B receptor-mediated contractions by enhancing translation in rat bronchi

**DOI:** 10.1186/1471-2466-6-6

**Published:** 2006-03-15

**Authors:** Bengt W Granström, Cang-Bao Xu, Elisabeth Nilsson, Petter Vikman, Lars Edvinsson

**Affiliations:** 1 Department of Medicine, Clinical sciences, Lund, Lund University, Sweden

## Abstract

**Background:**

Smoking is known to cause chronic inflammatory changes in the bronchi and to contribute to airway hyper-reactivity, such as in bronchial asthma. To study the effect of smoking on the endothelin system in rat airways, bronchial segments were exposed to DMSO-soluble smoking particles (DSP) from cigarette smoke, to nicotine and to DMSO, respectively.

**Methods:**

Isolated rat bronchial segments were cultured for 24 hours in the presence or absence of DSP, nicotine or DMSO alone. Contractile responses to sarafotoxin 6c (a selective agonist for ET_B _receptors) and endothelin-1 (an ET_A _and ET_B _receptor agonist) were studied by use of a sensitive myograph. Before ET-1 was introduced, the ET_B _receptors were desensitized by use of S6c. The remaining contractility observed was considered to be the result of selective activation of the ET_A _receptors. ET_A _and ET_B _receptor mRNA expression was analyzed using real-time quantitative PCR. The location and concentration of ET_A _and ET_B _receptors were studied by means of immunohistochemistry together with confocal microscopy after overnight incubation with selective antibodies.

**Results:**

After being cultured together with DSP for 24 hours the bronchial segments showed an increased contractility mediated by ET_A _and ET_B _receptors, whereas culturing them together with nicotine did not affect their contractility. The up-regulation of their contractility was blunted by cycloheximide treatment, a translational inhibitor. No significant change in the expression of ET_A _and ET_B _receptor mRNA through exposure to DMSO or to nicotine exposure alone occurred, although immunohistochemistry revealed a clear increase in ET_A _and ET_B _receptors in the smooth muscle after incubation in the presence of DSP. Taken as a whole, this is seen as the presence of a translation mechanism.

**Conclusion:**

The increased contractility of rat bronchi when exposed to DSP appears to be due to a translation mechanism.

## Background

Globally nearly 5 million deaths per year, as well as 12% of the deaths of people over 30 years of age are attributable to smoking, which makes smoking one of the world's most important health issues [1]. Cigarette smoke is known not only to cause chronic obstructive pulmonary disease, cancer, chronic bronchitis and asthma generally but also to cause suboptimal lung growth during the preadolescent and adolescent years [2]. It is a composite of irritant molecules, including acetaldehyde, hydroquinone, formaldehyde, benzo [*a*]pyrene, cresol, nicotine, catechol, acrolein, coumarin, anthracene, nitrogen oxides, and heavy metals [3]. Cigarette smoke causes rapid cell proliferation in the small airways and the associated pulmonary arteries [4].

Endothelin-1(ET-1) is the most potent vasoactive peptide described to date [5] and appears to have an important role in the regulation of pulmonary functions. It is synthesized, stored, released and metabolized in the lungs of many species including rat [6] and man [7], suggesting it to have a role both in normal physiology and in pathophysiological processes. The responses to ET-1 are mediated through endothelin type A (ET_A_) and type B (ET_B_) receptors. ET-1 has a similar affinity for ET_A _and for ET_B _receptors. In the tracheal smooth muscle and peripheral lung tissue of the rat ET_A _and ET_B _receptors are found in approximately equal numbers [8,9], whereas in the smooth muscle of the human bronchial airway ET_B _receptors predominate [10]. Both ET_A _and ET_B _receptors are present on smooth muscle cells of the airways, where they mediate strong contractions, although some ET_B _receptors are present on the airway epithelium as well, where they can induce relaxation through the release of nitric oxide [11]. ET-1 acts as a co-mitogen together with such factors as epidermal growth factor [12] It also causes increased secretion from both mucous and serous cells [13].

BQ-610, a selective ET_A _receptor antagonist, blocks mitogenesis induced in rat airways by cigarette smoke [14] and pretreatment by Bosentan, a nonselective endothelin receptor antagonist, inhibits the eosinophilic inflammatory response to Sephadex in BALF and in lung tissue [15]. The plasma endothelin level in humans is increased after smoking [16]. Pro-inflammatory mediators such as tumor necrosis factor-α (TNF-α) and interleukin-1β (IL-1β) have been found to promote increased production of endothelin-1 in guinea-pig cultured tracheal airway epithelial cells [17] and in murine tracheal segments. IL-1β up regulates the mRNA expression for ET-1 in mouse airways in which the epithelium is intact [18]. After treatment of human temporal arteries by IL-1β, a pro-inflammatory cytokine, both maximal contraction and potency mediated by ET_B _receptors have been found to increase [19].

The present study examines functional changes in bronchial ET receptors caused by smoking particles and investigates the underlying mechanisms causing these changes.

## Methods

### Tissue preparation and organ culture

Male Sprague Dawley rats (body weight 250 g, M&B, Denmark) were acclimatized for a week under standardized temperature (21–22°C), humidity (50–60%) and light (12:12 light-dark) conditions in the Animal Department of Wallenberg center in Lund. The rats were killed by CO_2 _and were exsanguinated. The lungs were immersed in cold buffer solution (composition, see below) and the bronchi were freed of adhering lung tissue down to the third generation (0.5 mm) by dissection under a microscope. Circular segments were cut from the bronchi with a diameter of 0.5–1 mm.

The bronchial segments were placed individually in wells containing DMEM, DMSO-solution (composition, see below) or nicotine and were put in an incubator for 24 hours. Incubation was carried out at 37°C in humidified air containing 5% CO_2_. After incubation the bronchial segments were divided into three groups for functional myograph studies, for immunohistochemistry and confocal microscopy, and for real-time quantitative RT-PCR for mRNA expression – such that each animal contributed one bronchial segment to each of the three studies carried out. The protocol was approved by the animal ethics committee at Lund University (M-120-01).

### Functional studies

Each bronchial segment was mounted on two L-shaped metal prongs. One prong was connected to a force displacement transducer attached to a computer for continuous registration of isometric tension and the other to a displacement device. The segment was immersed in small (2.5 ml) temperature-controlled (37°C) tissue baths containing a bicarbonate-based buffer solution (see below). The solution was equilibrated by 5% CO_2 _in O_2_, resulting in a pH of 7.4. Initially the bronchial segments were allowed to stabilize for 60 min under a tension of 0.8 mN. The pretension employed was chosen on the basis of pretension-contraction curves in Ca^++ ^free and Ca^++ ^containing solution as described earlier [20] and as later modified for bronchial ring segments [Granström, unpublished]. The contractile ability of each segment was first examined by exposure to a potassium-rich (60 mM) buffer solution (for composition, see below), which produced a maximum contractile effect at this concentration. Maximum contraction was reached within a few minutes. The potassium solution was washed out then by the buffer solution. The individual segments were only used for further studies if two strong (>1 mN) reproducible contractions (variation <10%) could be elicited. The contraction induced by the K^+^-solution (60 mM) was used as a contractility reference and its maximum was defined as 100%. The epithelium was adjudged to be intact since previous studies of endothelin receptors had not shown any effect of the presence or absence of the epithelium [21]. At a point 30 minutes before cumulative concentrations of sarafotoxin (S6c, a specific ET_B _receptor agonist) or ET-1 were administered, 3 μmol of indometacin and 100 μmol of L-NG-monometylarginin (L-NMMA) were added to block the modifying effects of epithelial prostaglandin and NO release [22]. The segments were allowed to stabilize at each contraction level before a higher concentration of the agonist was added. When the maximum concentration and contraction that S6c produced was reached, the contractile response was allowed to fade away during a 1-hour period. Thereafter the tissue bath solution was exchanged and new substances were added, including S6c. No contraction was observed then, indicating a total desensitizing of ET_B_. The subsequent administration of ET-1 in cumulative concentrations resulted in concentration-effect curves mediated only by contractions via ET_A _receptors [18]. The maximal contractile force was tested at the end of each experiment by use of acetylcholine (10^-3 ^M).

Contractility was tested in fresh bronchial segments and in control segments incubated together with DMSO, nicotine or DSP for 24 hours. To confirm that up-regulation was caused by the translational mechanism, bronchial segments were incubated for 24 hours in DSP solution with and without cycloheximide. Acetylcholine contraction was used as a reference since carbachol induced contractions appear to be more stable under various conditions of incubation than KCl contractions are [23,24].

### mRNA quantification

#### Total RNA isolation and reverse transcription into cDNA

Here the bronchial segments were taken from incubation wells and any residues of surrounding pulmonary tissue were carefully dissected away. The segments were then snap frozen in liquid nitrogen for RNA isolation and transcription was carried out. None of these segments had been used for functional experiments prior to this preparation. The segments were homogenized in 1 ml of the RNApro™solution (Q-BIOgene, CA, USA) by use of a FastPrep^® ^instrument (Q-BIOgene, CA, USA). Total RNA was extracted following a protocol from the FastRNA^® ^Pro kit supplier. Reverse transcription of total RNA to cDNA was carried out using the Gene Amp RT kit (PE Applied Biosystems) in a Perkin-Elmer 2400 PCR machine at 42°C for 30 min.

#### Quantification of the expression of ET-1, ET_A_, ET_B _receptor mRNA and ET-1 mRNA

Real-time quantitative RT-PCR was performed here by the GeneAmp SYBR Green PCR kit (PE Applied Biosystems, USA) in a Perkin-Elmer real-time PCR machine (GeneAmp 5700 sequence detection system). The system automatically monitors the binding of a fluorescent dye to double-strand DNA by real-time detection of the fluorescence present during each cycle of PCR amplification. Specific primers for rat ET-1, ET_A_, ET_B _receptors were designed as below:

ET-1 forward: 5'-TTTTGAAGACCGCGCTGAG-3'

reverse: 5'-GGTTGCTCTGATCGCCTCTG-3'

ET_A _receptor forward 5'-GTCGAGAGGTGGCAAAGACC-3'

reverse 5'-ACAGGGCGAAGATGACAACC-3'

ET_B _receptor forward: 5'-GAT ACG ACA ACT TCC GCT CCA-3'

reverse: 5'-GTC CAC GAT GAG GAC AAT GAG-3'

The housekeeping gene, Elongation factor-1 (EF-1), mRNA, which is continuously expressed at a constant level in the cells, was compared in a pilot study with the housekeeping gene β-actin by use of real-time PCR (data not shown). EF-1 was employed as a reference, but both showed the same degree of constancy in the tests. The rat EF-1 primers were designed as follows:

EF-1 forward: 5'-GCA AGC CCA TGT GTG TTG AA-3'

reverse: 5'-TGA TGA CAC CCA CAG CAA CTG-3'

The PCR reaction, performed in a 50 μl volume, started at 50°C for 2 min, followed by 95°C for 10 min, then 40 PCR cycles at 95°C for 15 sec and 60°C for 1 min. Dissociation curves were run after the real-time PCR, no non-specific amplification detected in the present study. Each of the primers was designed using the Primer Express 2.0 software (PE Applied Biosystems, USA) and was synthesized by GibcoBRL Custom Primers (Life Technologies, Inc., USA).

The gene IDs in the gene bank accession number were as follows:

ET-1 gene ID (NM_012548)

ETA gene ID (NM_012550)

ETB gene ID (NM_017333)

EF-1 gene ID (BC072542)

The PCR products of ET_A _(64 bp), ET_B _(86 bp) and EF-1 (96 bp) were visualized by agarose gel electrophoresis Electrophoresis and dissociation curves were used to verify the specificity of the PCR products.

To evaluate the amount of ET_A _or ET_B _receptor mRNA a sample contained, EF-1 mRNA was assessed in the sample simultaneously. The cycle threshold values of EF-1 mRNA were used as a reference to quantify the relative amounts of ET_A _or ET_B _receptor mRNA. The relative amount of mRNA was calculated for the cycle threshold values of ET_A _or ET_B _receptor mRNA in relation to the cycle threshold values of EF-1 mRNA in the sample.

### Immunohistochemistry

After incubation, the bronchial segments were placed on Tissue TEK (Gibco) and frozen. Three different samples were used in each group. The segments were sectioned into 8 μm thick slices in a cryostat. The primary antibodies used were rabbit antihuman ET_B _(IBL, 16207, diluted 1:400), goat anti human ET_A _(Santa Cruz Biotechnologies, sc-21194, diluted 1:100) and mouse anti rat smooth muscle actin (Serotec, MCA1905T, diluted 1:100). All dilutions were performed in PBS, using 10% fetal calf serum. The secondary antibodies employed were donkey anti mouse Cy™5 conjugated (JacksonImmunoResearch, 715-175-150, 1:100), donkey anti rabbit Cy™3 conjugated (JacksonImmunoResearch, 711-165-152, 1:100) and donkey anti goat Cy™2 conjugate (JacksonImmunoResearch, 705-225-003) in PBS. The antibodies were detected at the appropriate wavelength by confocal microscopy (Zeiss, USA), unspecific background being subtracted. Only secondary antibodies were used as a control. The absolute fluorescence intensity was measured with ImageJ [25]

### Solutions

(A) Standard buffer solution (mM): NaCl 119, KCl 4.6, CaCl_2 _1.5, MgCl_2 _1.2, NaHCO_3 _15, NaH_2_PO_4 _1.2, and glucose 5.5.

(B) 60 mM K^+ ^buffer solution: as above, but with substitution of equimolar amounts of NaCl containing KCl.

(C) Dimethyl Sulfoxide (DMSO).

(D) Three cigarettes (0.8 mg nicotine per cigarette) were "smoked" by a water aspirator, the smoke being directed through a cotton-wool filter. The smoke particles retained in the filter were dissolved in 1 ml DMSO. The DMSO-soluble smoke particle (DSP) preparations were analyzed by gas chromatograph-flame ionisation detection (GC-FID, Agilent 6890N, USA) on a 0.23 mm × 15 mm × 0.25 m DB-5MS capillary column (Agilent, USA). The GC-FID temperatures were programmed to increase by 5°C/min from 50°C, to 280°C and remain there for 3 min. The concentration of nicotine in DSP was calculated according to the standard nicotine peak value and area. After DSP preparations had been analyzed by gas chromatography, they were diluted by DMSO to standard nicotine content (0.11 mg/ml) and were used for the organ culture experiments. Pure nicotine was employed at a concentration of 0.10 mg/ml.

### Drugs

Sarafotoxin 6c and endothelin-1 were obtained from Auspep (Parkville, Australia) and L-NMMA, indometacin, acetylcholine and cycloheximide from Sigma (St. Louis USA). These agents were dissolved and further diluted in saline containing 0.1% bovine serum albumin (Beringwerke, Marburg, Germany) to avoid adhesion of peptides to the vials.

### Analysis

Contractile responses in the segments are expressed as the percentage of contraction induced by 60 mM K^+^. The E_max _values refer to the maximum contractile effect of an agonist. The pEC_50 _value (the negative logarithm of the molar concentration that produced a half-maximum contraction) was calculated for the concentration above and below the midpoint of the concentration-response curve using a straight-line equation.

### Statistics

Students' unpaired t-test was used for the molecular studies. A p-value of <0.05 was considered as significant.

## Results

### Contractile responses

**Figure 1:**
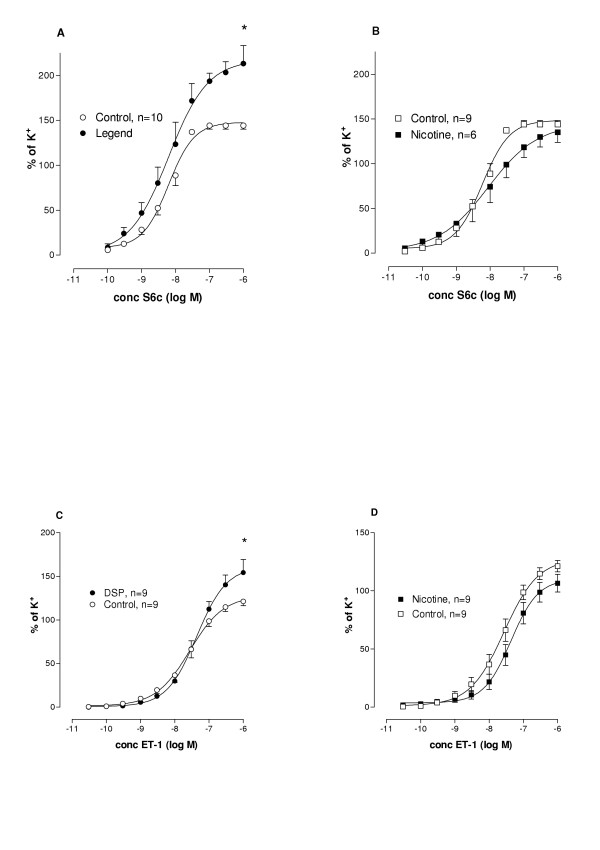
**ET-1 and S6c contraction after 24 H of DSP or nicotine incubation**. Bronchial smooth muscle cell responses to the concentration-dependent application of sarafotoxin 6c (S6c) or endothelin-1 (ET-1) following incubation for 24 h together with DSP (A, C) or nicotine (B,D) as compared with incubation control (DMEM). Mean values with S.E.M. are given. Unpaired t-tests were used to compare the groups in terms of maximum induced contraction; * P < 0.05.

In bronchial smooth muscle a significant increase was obtained (p < 0.05) in the maximum contractile response to S6c following its incubation together with DSP (Figure 1A) but not with nicotine.

ET_A _receptor contractility was tested after desensitization of ET_B _(see MATERIALS AND METHODS/*Functional studies*). The contractions produced by ET-1 that follow have been found to be mediated by ET_A _receptors and can be blocked by a selective ET_A _antagonist [8]. The contractions were significantly stronger in DSP treated segments (p < 0.05) but not in segments exposed to nicotine (Figure 1C and 1D). DMSO at the concentration employed did not affect contractility (not shown).

Segments, that were maximally contracted by ET-1 through use of the ET_A _receptors, showed no further contraction when acetylcholine (10^-3 ^M) was added.

**Figure 2:**
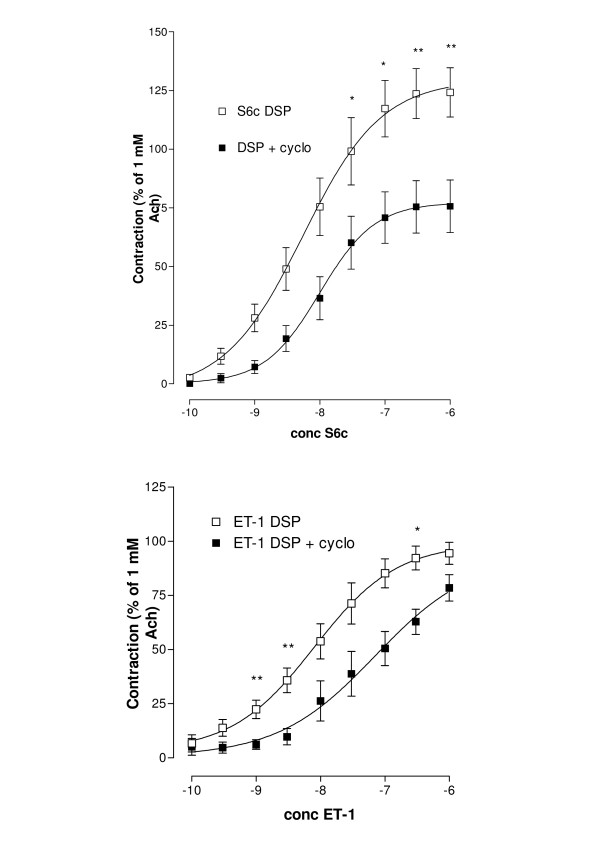
**Translational inhibitor (cycloheximide) effect on DSP led to changes in ET-1 and S6c contractility**. Rat bronchial segments were incubated for 24 hours in DSP in two groups. In the one group a translational inhibitor (cycloheximide, 10 μg mL^-1^) was added. Two-way  Anova with Bonferroni post test was used to compare contraction at different  concentrations of agonist. * P < 0.05.

Adding cycloheximide (10 μg mL^-1^) to segments incubated with DSP for 24 hours showed a significant reduction of S6c and to a less extent ET-1 contractility (Figure 2).

### mRNA studies

**Figure 3:**
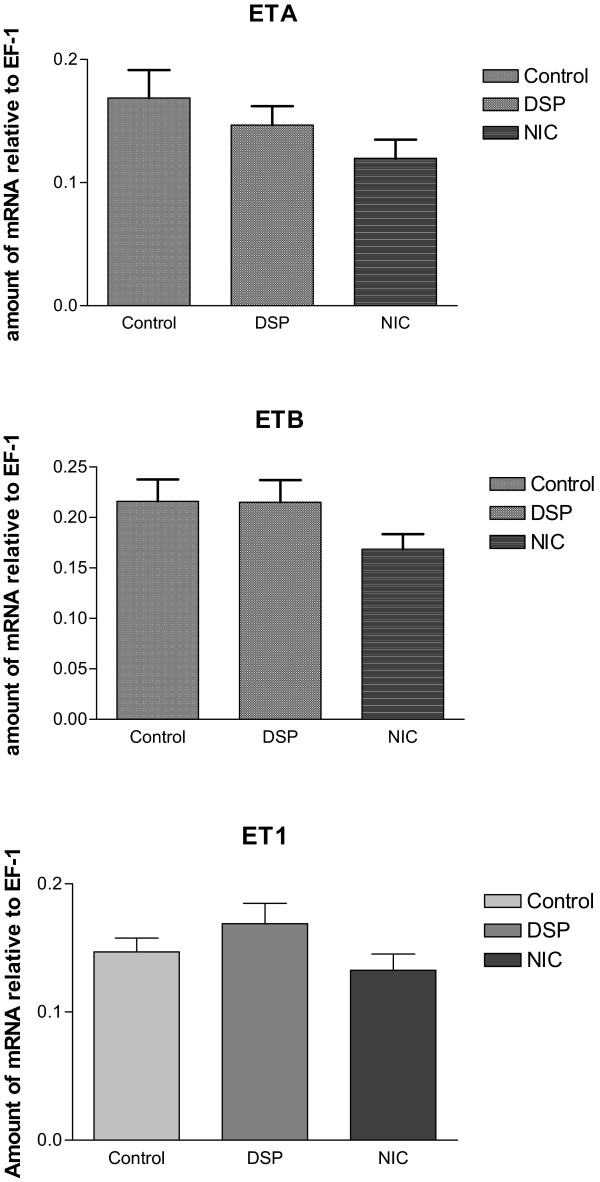
**mRNA expression of ET_A _receptors (A), ET_B _receptors (B) and ET-1 in the bronchi**. The mRNA expression of ET_A _and ET_B _receptors and ET-1 in the bronchi was quantified by means of real-time PCR. Each data point was derived from results for at least 3 segments. No differences in the expression level after DSP or nicotine treatment were found as compared with controls (DMSO), (p > 0.05).

The amount of mRNA for ET_A _and ET_B _receptors in the smooth muscle as well as the ET-1 mRNA were quantified with the real-time PCR. No significant differences between the control (DMSO), the DSP- and the nicotine-treated groups (p > 0.05) (Fig. 3A–C).

### Immunohistochemistry and confocal microscopy

**Figure 4:**
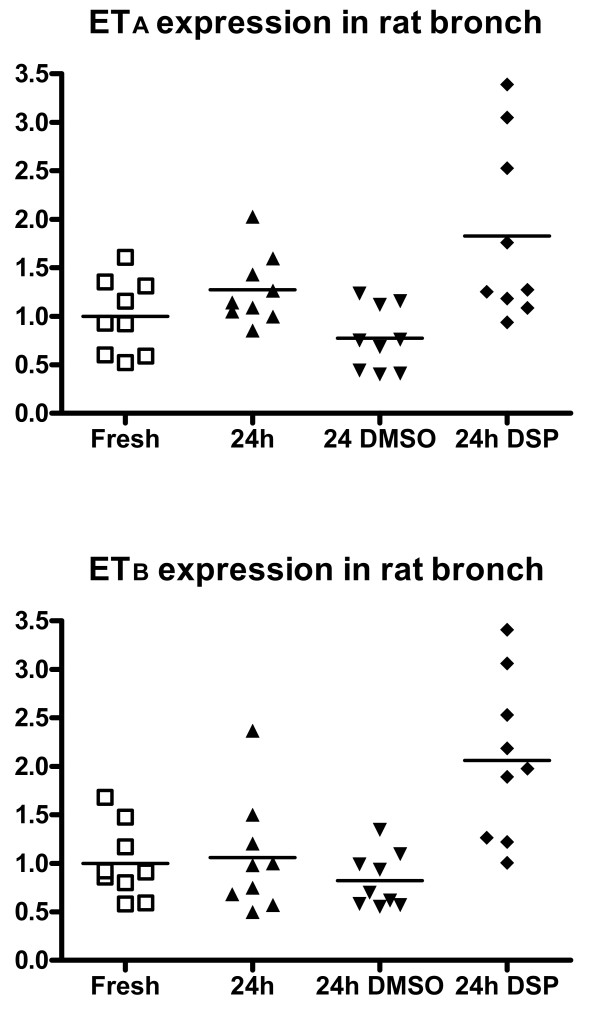
A and B; Quantitative image analysis of the bronchi for receptor protein expression under differing experimental conditions. ET_A _and ET_B _receptor protein showed a significant increase (p < 0.05) in DSP treated as compared with fresh bronchi, 24 h and 24 h+DMSO treated bronchi.

Immunohistochemistry revealed no up regulation of the amount of ET_A _or ET_B _receptor protein present in smooth muscle sections of the bronchi after incubation in the DMEM medium or in the DMEM medium together with DMSO as compared with the fresh control group (0 hours). ET_A _and ET_B _receptor protein staining revealed an up-regulation of the amount of protein in the DSP treated group as compared with the other groups (Fig 4A and 4B).

Image analysis of each of the segments showed no difference between the groups in the amount of actin staining (not shown) but, there was a significantly stronger ET_A _and ET_B _receptor protein expression (p < 0.05) (Figure 4B, 4C).

## Discussion

This is the first study to show lipid-soluble smoking particles "DSP" to cause a bronchial hyperresponsiveness to endothelin-1, a strong airway constrictor, and to S6c, a specific ET_B _receptor agent. Since no change in mRNA was found, except in protein expression, the effect can be considered to be mediated by an increase of the number endothelin receptors via translation. Smoking has been shown in the long run to result in airway distortion and an increase in airflow resistance, and to lead to the airways becoming muscularized and fibrotic. Similar changes can be observed after several decades of asthmatic disease. More recently, it has been shown that in childhood asthma such changes occur early.

Previous studies of the endothelin system have led to differing results. We found bronchial biopsies from patients with asthma and chronic airway obstruction to show significantly higher levels of endothelin ET_B _receptor mRNA than of endothelin ET_A _receptor mRNA [26]. Inflammation induced in the human temporal by the pro-inflammatory cytokine interleukin-1beta (IL-1beta) artery has been found to result in an increase in maximal contraction and potency in response to S6c but not to any change in the ET_A_/ET_B _receptor mRNA ratio [19]. The explanation suggested was that IL- 1beta may further stimulate translation of the mRNA to active receptors. The hyperreactivity could also be a function of the length of exposure to the pro-inflammatory stimuli. Culturing murine tracheal segments in the presence of IL-1β has been found to attenuate the maximal contraction produced by ET_B _receptors and downregulated expressions of the ET_B _receptor mRNA. This was first observed after 2 days of culture, reduction being maximal at 4 days [18]. Bronchial smooth muscle from rats exposed to cigarette smoke for two weeks was found to show hyperreactivity to acetylcholine but no depolarization due to high K+ level, this being explained in terms of increased expression of the RhoA-protein [27].

Another well-known feature of pulmonary inflammation is epithelial damage. Early asthmatic inflammation has been found to cause disruption of the epithelium [28]. In smoking, the respiratory epithelium is damaged or missing. In the presence of DSP, arterial endothelium lost its attachment and functional tests showed reduced dilation [29]. The clearance of ET-1 in the airways through the ET_B _receptors on the epithelial cells is probably impaired, leading to enhanced access of ET-1 to underlying bronchial smooth muscle cells [30]. The production of relaxant factors such as nitric oxide through ET-1 binding to receptors on the epithelial cells is also compromised [11].

In the present study, involving use of rat bronchi, we found the maximal contractile response to ET_A _and ET_B _receptors to be significantly augmented by the presence of DSP but not by nicotine incubation. In preliminary tests in rat mesentery artery, the water-soluble part of the smoking particles was not found to alter ET_B _receptor expression (data not shown). The identity and the characteristics of the receptors have been analyzed in detail previously [10]. Further support for the hypothesis of an endothelin receptor protein increase was obtained by immunohistochemistry. This revealed a slight increase in ET_A _and a strong and significant increase in ET_B _receptors after culturing together with DSP. These up-regulated receptors were located in the smooth muscle cell layer. We have shown previously that eosinophilic inflammation induced in vivo by Sephadex results in ET_B _receptor up regulation, both functionally and at the mRNA level [21]. We did not observe any further change in mRNA expression for ET_A _or ET_B_. However, there have been earlier reports of a decrease in ET_B _receptor mRNA expression in mouse airways after long-term treatment by IL-1β [18]. To explain our own results further, we used confocal microscopy to indicate whether the amount and the localization of receptor protein were changed. This revealed an up-regulation of ET_A _and ET_B _receptors in the smooth muscle cells. *De novo *transcription and translation via protein kinase C [31] and mitogen-activated protein kinases [32,33] have been shown to occur after organ culture of arterial segments.

In vascular studies, cytokines derived from inflammatory cells during airway inflammation, could lead both to increase in the level of ET-1 and an up regulation of endothelin receptors that synergistically enhance the contractility of the smooth muscle of the airways. Use of an endothelin antagonist is already an established treatment for pulmonary hypertension[34].

Most ET-1 is secreted abluminally towards the smooth muscle cells. A small part of ET-1 can be measured in plasma and smoking leads to raised level of plasma ET-1 [16]. In our study we did not measure any ET-1 level but the mRNA expression of ET-1 was not altered. Thus, if up-regulation of ET-1 was present in our study, it must be explained as due to a translational mechanism, since the mRNA was unaltered. Raised levels of ET-1 may cause a raised consumption of ET_B _receptors, i.e. an increased turnover, explaining varying results in different studies

In order to test whether nicotine *per se *could be involved in this process, it was included in our study. We found no increase in ET_A _or ET_B _receptor expression after nicotine exposure. This is in agreement with the finding elsewhere that 24 hours of nicotine incubation of human coronary artery endothelial cells had no effect on the expression of ET-1 [35].

## Conclusion

Although the mechanisms causing the up-regulation of ET_A _and ET_B_receptors in airways, through *de novo *synthesis, are not known, the phenomenon has been studied in detail for blood vessels. Putatively, a regulatory mechanism similar to that in blood vessels could conceivably take place in bronchi but further study would be needed to determine this. Other mechanisms for the regulation of ET-receptors in airway inflammation may hypothetically occur via such cytokines as tumor necrosis factor-α and interleukin-1α. Our group has demonstrated that such a phenomenon can occur in vascular smooth muscle cells [36,37]. Study of such regulatory mechanisms in the airways can be regarded as extremely important.

## Competing interests

The author(s) declare that they have no competing interests.

## Authors' contributions

BWG participated in the design of the study and played a major role in acquisition, analysis and interpretation of data and drafted the manuscript. CBX performed RT-PCR analysis, participated in drawing the figures and contributed to writing the manuscript. EN helped to perform the contraction studies. PW was responsible for implementation of the immunohistochemistry with use of confocal microscopy and for the drawing of these figures. LE contributed to the design of the study, interpretation of results and writing of the manuscript. Each of the authors read and approved the final manuscript.

## Pre-publication history

The pre-publication history for this paper can be accessed here:



## Figures and Tables

**Figure 5:**
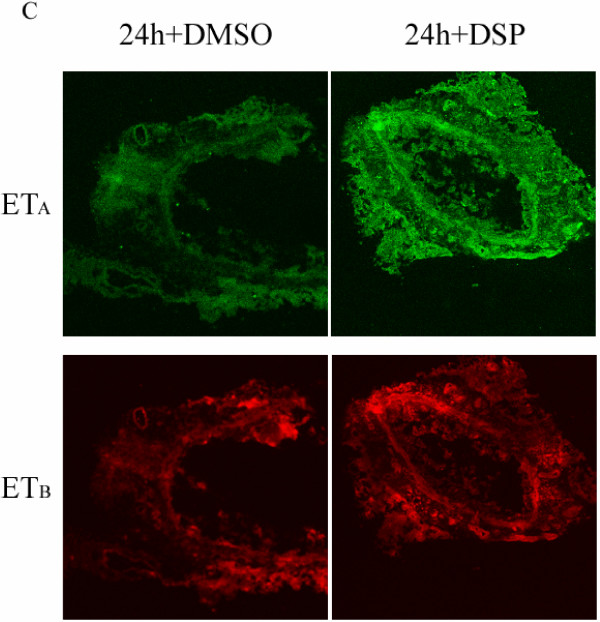
**Immunostaining of ET_A _and ET_B _receptor protein in bronchi**. Figure C. Example of immunostaining of ET_A _and ET_B _receptor protein after 24 h of culture together with DMSO as compared with 24 h of culture together with DSP.

**Table 1:** Maximal contractile responses after DSP or nicotine incubation. The maximal contractile responses (% of maximal contraction to 60 mM potassium) and pEC_50 _of endothelin-1 and sarafotoxin 6c in bronchial segments from 24 hour DSP or nicotine incubation as compared with control segments The values are given as means ± S.E.M., n = 6–10. Unpaired t-tests were used to compare the groups in terms of maximum induced contraction; ^#^* P < 0.05.

**Rat Bronchi**		
	Maximal contraction	pEC_50_
**Endothelin-1**		
Control	121.21 ± 4.84^#^	7.69 ± 0.49
DSP	154.24 ± 14.88^#^	7.43 ± 0.35
Nicotine	106.49 ± 7.53	7.39 ± 0.13
**Sarafotoxin6c**		
Control	144.25 ± 4,01*	8.24 ± 0.08
DSP	213.39 ± 20.03*	8.21 ± 0.17
Nicotine	135.01 ± 11.25	8.24 ± 0.35
